# Oxytocin and the Neurobiology of Prosocial Behavior

**DOI:** 10.1177/1073858420960111

**Published:** 2020-09-26

**Authors:** Nina Marsh, Abigail A. Marsh, Mary R. Lee, René Hurlemann

**Affiliations:** 1 Department of Psychiatry, Carl von Ossietzky University Oldenburg, Oldenburg, Lower Saxony, Germany; 2 Department of Psychology, Georgetown University, Washington, DC, USA; 3 Clinical Psychoneuroendocrinology and Neuropsychopharmacology Section, National Institute on Drug Abuse Intramural Research Program, Baltimore, MD, USA; 4 National Institute on Alcohol Abuse and Alcoholism Division of Intramural Clinical and Biological Research, National Institutes of Health, Bethesda, MD, USA; 5 Research Center Neurosensory Science, Carl von Ossietzky University Oldenburg, Oldenburg, Lower Saxony, Germany

**Keywords:** oxytocin, prosocial behavior, altruism, care, social neuroscience

## Abstract

Humans are an unusually prosocial species, who engage in social behaviors that include altruism—whereby an individual engages in costly or risky acts to improve the welfare of another person—care, and cooperation. Current perspectives on the neurobiology of human prosociality suggest that it is deeply rooted in the neuroendocrine architecture of the social brain and emphasize the modulatory role of the neuropeptide hormone oxytocin. In this review, we provide a conceptual overview of the neurobiology of prosocial behavior with a focus on oxytocin’s modulatory role in human prosociality. Specifically, we aim to encourage a better understanding of the peptide’s susceptibility to diverse factors that produce heterogeneity in outcomes and the resulting methodological implications for measuring the behavioral effects of oxytocin in humans. After providing an overview of the state-of-the-art research on oxytocin’s exogenous use, we elaborate on the peptide’s modulatory role in the context of care-based altruism, cooperation, and conflict and discuss its potential for therapeutic interventions in psychiatric disorders characterized by social dysfunction.

## Introduction

**Figure 1.**
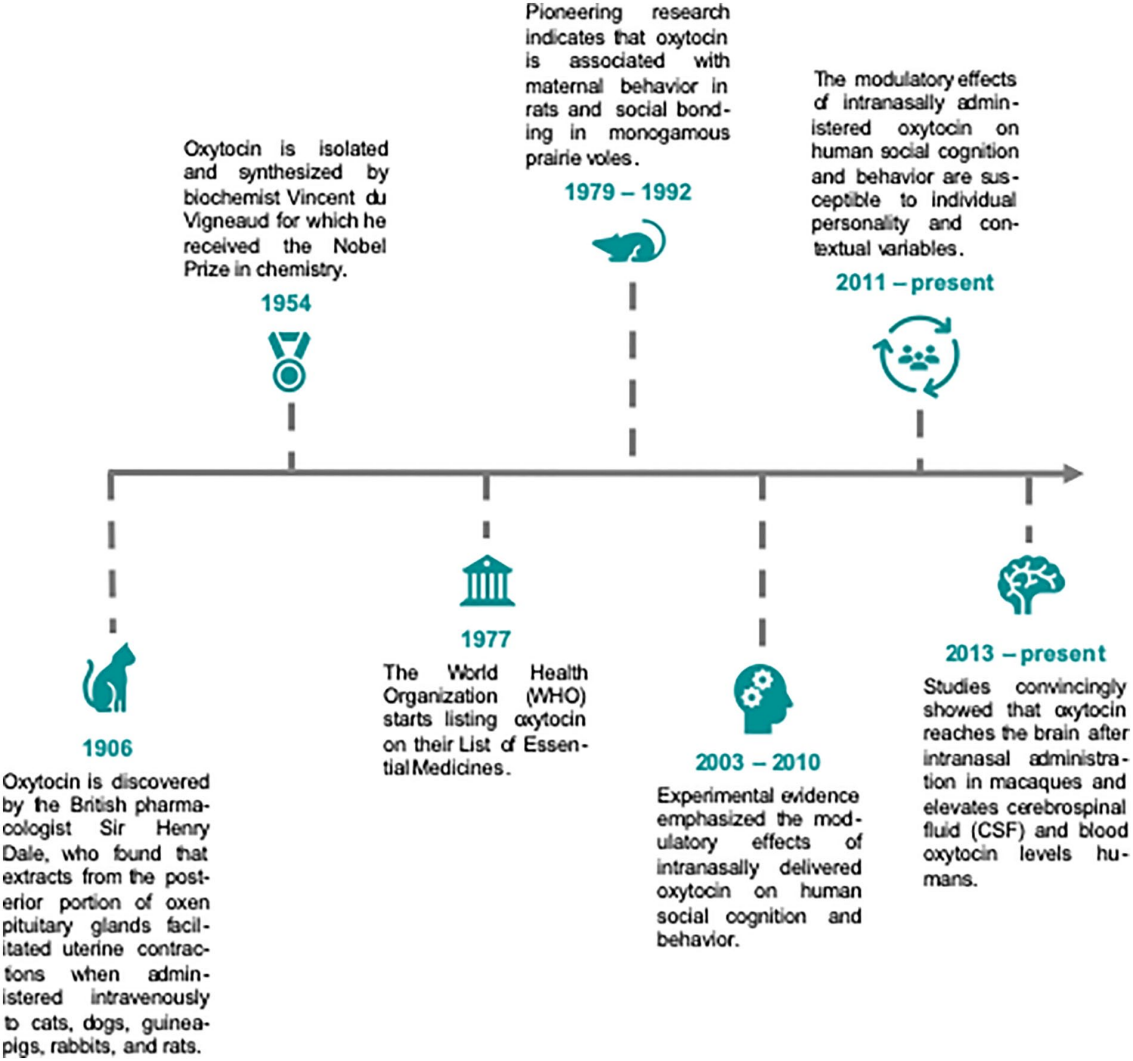
A brief overview of the history of oxytocin. The hypothalamic peptide hormone oxytocin has played an important role in medicine with major highlights including the identification of its involvement in parturition and lactation. Since these initial discoveries, oxytocin has become one of the most highly researched agents in the mammalian nervous system due to its role as a neuromodulator and neurotransmitter. Experimental evidence from animal and human studies convincingly demonstrated that oxytocin not only regulates reproductive functions but is also centrally involved in the modulation of social behavior and cognition.

Humans are notable for the wide scope of their prosocial behavior (Silk and House 2011), which is subserved by neuroendocrine systems originally adapted to enable reproduction and motivate related parental care of the offspring and attachment. A multitude of studies in human and nonhuman animals have linked the highly evolutionarily conserved neuropeptide oxytocin (OXT) (Fig. 1) to a rich repertoire of social behaviors (Donaldson and Young 2008; Hurlemann and Scheele 2016; Marsh 2016a). OXT is well known for its functions as a neurotransmitter, neuromodulator and neurohormone: in the brain, endogenous OXT is synthesized in the paraventricular, supraoptic, and accessory nuclei of the hypothalamus, which send projections to the posterior pituitary gland whereupon they secrete OXT into systemic circulation. The dynamics of OXT release depends on the nature of the stimulus—for example, reproductive or stressful—promoting this release. Peripherally, OXT exerts a neurohormonal role in inducing uterine contraction during parturition and milk let-down during lactation (Carter 1998; Keverne and Kendrick 1992). Intracerebrally, OXT release may result from a combination of two different release modes, which, together, may affect all major forebrain regions: synaptically as a neurotransmitter (wiring transmission) and non-synaptically as a neuromodulator (volume transmission). The latter refers to the diffusion of OXT molecules into the extracellular milieu, where they interact with available OXT receptors in a radius that spans up to 120 μm (Chini and others 2017). Humans possess only one type of OXT receptor (OXTR), neuronal and glial expression of which is regulated—most likely in a region-specific manner—via endogenously and exogenously triggered mechanisms, including epigenetic modification, ligand availability, changes in hormonal status (e.g., along the estrus cycle), age, stage of development, and acute or chronic exposure to stressors (Kraaijenvanger and others 2019). Such regulations are thought to be etiologically relevant for the psychophysiology of psychiatric conditions and may thus provide potential treatment targets. For example, in rodents, the OXTR, a 7-transmembrane G protein-coupled receptor capable of binding to either Gαi or Gαq proteins, activates a set of intracellular signaling cascades involving cytoplasmic and nuclear targets to induce protein synthesis, which has been shown to mediate the anxiolytic effects of OXT (Martinetz and others 2019) and improve memory formation (Tomizawa and others 2003).

In humans, the intracellular signaling pathways engaged by OXT remain poorly understood. Due to the lack of a CNS-penetrating PET (positron emission tomography) ligand, the in vivo regional distribution of OXTR is also enigmatic. However, analysis of postmortem OXTR mRNA distribution patterns may provide a proxy. Using this method, OXTR was found to be enriched—in high co-expression with several dopaminergic and muscarinic acetylcholine genes—in olfactory and subcortical regions (Quintana and others 2019). Another source of variability is genetic variation in the *oxtr* gene (mostly intronic single-nucleotide polymorphisms [SNPs]) and other OXT-pathway genes, which has been associated with a multitude of social-emotional phenotypes, although replication of initial findings is often complicated by factors such as gender, culture, and early environment (Feldman and others 2016). While experimental manipulation of endogenous OXT release via physiological interventions (Jong and others 2015) or pharmacological (Gulliver and others 2019), optogenetic (Knobloch and others 2012) or chemogenetic (Grund and others 2019) manipulations is an important target of current research, seminal studies have shown that intranasal, that is, exogenous administration of synthetic OXT induces subtle changes in social behaviors; these alterations have often been described as tendencies toward increased sociality, including trust (Baumgartner and others 2008; Kosfeld and others 2005), empathy (Hurlemann and others 2010), approach (Preckel and others 2014; Scheele and others 2012), and altruism (Marsh and others 2015), all of which may be mediated by OXT’s anxiolytic and antistress effects in the first place (Eckstein and others 2016; Heinrichs and others 2003). This anxiolytic, antistress and prosocial profile of oxytocin is further enhanced by findings from neuroimaging studies reporting that OXT consistently targets reward-related (Donaldson and Young 2008; Scheele and others 2013; Skuse and Gallagher 2009; but see Striepens and others 2014) and fear-related neurocircuits (Kirsch and others 2005; Meyer-Lindenberg and others 2011). As a consequence, there is a growing interest in translating OXT neuroscience into a potential treatment for psychiatric conditions ranging from anxiety to autism spectrum disorders, depending on the specific dysfunction that is targeted with OXT.

Accumulating evidence, however, indicates that the effects of OXT on prosocial behavior are highly susceptible to variation due to individual and contextual variables, that is, OXT does not invariably facilitate positive social behaviors but may also produce protective or even defensive-aggressive responses. For example, it has been found that OXT increases envy and gloating (Shamay-Tsoory and others 2009), decreases the tendency to cooperate in individuals with borderline personality disorder (Bartz and others 2011), and may facilitate favoring in-group members, or even out-group derogation (De Dreu and others 2010; 2011; Van IJzendoorn and Bakermans-Kranenburg 2012).

These opposing effects indicate that OXT does not exclusively promote proximal prosocial behaviors and also emphasizes the peptide’s vulnerability to context- dependent factors. Thus, OXT-studies must be designed with appropriate methodological rigor in order to avoid the misinterpretation of data. The rationale of this review is to encourage a better understanding of OXT as a neuropeptide that does not uniformly or simply promote prosocial behaviors. Given the complex neurobiology of the OXT-system, including oxytocinergic pathways, local release patterns, and OXT receptor distribution in the brain, as well as intraneuronal OXT receptor signaling (Grinevich and Neumann 2020), the magnitude and the direction of OXT effects on neural and behavioral responses is not based on simple formula resulting in positive effects across all individuals and all situations (Andari and others 2018; Hurlemann 2017). The design and interpretation of OXT-research requires a more nuanced understanding of both situational factors, for example, the framing of an experimental paradigm (Marsh and others 2015), as well as individual variations in personality traits and prosocial motivations, including trust, affiliative motivation, early-life adversity, or empathy (Declerck and others 2020; Marsh and others 2017; Van IJzendoorn and others 2011). Given its sensitivity to individual personality and external variables, certain methodological considerations, for example, regarding sample size, population characteristics, and the use of social message frames, are essential for conducting—and independently replicating—OXT experiments. Because the majority of human trials use the intranasal route of administering OXT to experimentally modulate neural and behavioral outcomes, we first review current perspectives on this route of administration with its potential for “nose-to-brain” delivery of OXT and reflect on the methodological implications for experimental OXT-research. In the second section, we focus on OXT’s modulatory role in human prosociality and provide a more detailed overview of the peptide’s susceptibility to various context-dependent and personality-specific influences. In the third part, we outline OXT’s potential role in the development of novel clinical interventions and the implications for treating psychiatric disorders characterized by social dysfunction.

## Current Perspectives on Exogenous Oxytocin in Experimental Trials

### Using Exogenous OXT to Modulate Endogenous OXT Activity

The most precise measurements of OXT’s effects on mammalian neural activity and behavior are obtained through invasive techniques that include intracerebral microdialysis, targeted delivery of OXT antagonists, gene knockout, and viral gene transfer. However, the invasive nature of these techniques makes them impractical for human research. Thus, the effects of administered OXT in humans are typically examined by measuring changes in OXT concentrations in urine, saliva, blood, or cerebrospinal fluid (CSF) (as well as by defining relevant behavioral, physiological, and neuroimaging endpoints). Intravenously administered OXT has been shown to induce behavioral effects (Hollander and others 2003), but several studies indicate that only a small fraction of the peptide passes the blood-brain barrier (BBB) (Kang and Park 2000; Lee and others 2018; Mens and others 1983;). OXT cannot be administered by mouth due to degradation by enzymes in the gastrointestinal tract and by first-pass metabolism. Thus, the intranasal route is preferred, as it bypasses both these pathways.

Methodologically, intranasal OXT administration is the most direct and non-invasive way to assay the central actions of OXT in humans (Meyer-Lindenberg and others 2011). Studies typically involve a three-step design: the administration of 24 international units (IU) of intranasal OXT, a 45-minute waiting period, and a time window of active OXT effects during experimental testing (Guastella and others 2013). After intranasal administration, OXT may pass into the systemic circulation via absorption by the capillaries in the nasal mucosa. Once in the systemic circulation, it may cross the BBB and enter the CSF and brain (Beard and others 2018; Lee and others 2018; Martins and others 2020; Striepens and others 2013). Alternatively, or in addition, intranasally delivered OXT may enter the brain parenchyma and extracellular fluid directly, bypassing the BBB. In a nonhuman primate study administering labelled OXT intranasally and intravenously, Lee and colleagues (2020) recently demonstrated that OXT delivered intranasally (not intravenously) reached brain regions that lie along the trajectory of the olfactory and trigeminal nerves in concentrations adequate to activate the OXT receptor. With the intravenous condition as a control, results from this study indicate that intranasal OXT bypasses the BBB (Table 1). Similar results were obtained after intranasal administration of interferon-β to nonhuman primates (Thorne and others 2008). Variation in brain OXT penetration may result from differences between monkeys in OXT receptor expression in some brain regions. Also, the interval between administration and perfusion was long considering the half-life of OXT. Further studies over a shorter time course are warranted to determine whether intranasal administration leads to reliable delivery of OXT to these brain regions. Relevant to the small percentage of administered OXT that crosses the BBB, recent studies have identified a receptor for advanced glycation end product (RAGE), which transports OXT across the endothelium of the gastrointestinal tract (Higashida and others 2017) and the BBB (Higashida and others 2019; Yamamoto and Higashida, 2020). Furthermore, it has been shown that measuring biologically relevant changes in salivary OXT can be observed using a sensitive enzyme immunoassay (EIA) (Carter and others 2007).

**Table 1.** Regional Distribution of Endogenous Brain Oxytocin Levels (nM) in Rhesus Macaques Prior to Exogenous Oxytocin Administration.^a^

	80 IN		40 IN		80 IV	
Monkey ID # (Sex)	ID #2 (M)	ID #4 (M)	ID #1 (F)	ID #5 (F)	ID #3 (F)	ID #6 (F)
Cerebellum	ND	<1	12	<1	ND	+
Brainstem	28	19	40	51	60	20
Striatum	9	97	3	7	79	86
Amygdala	32	131	NM	214	25	40
Thalamus	6	17	24	40	21	155
Visual cortex	ND	ND	ND	ND	ND	ND
Insular cortex	ND	<1	NM	2	1	ND
dPFC	ND	1	ND	1	2	ND
mPFC	ND	<1	<1	<1	ND	ND
OFC	2.2	9	1	5	2	ND
Hypothalamus	368		32,000		895	
Hippocampus	15		5		5	

dPFC = dorsal prefrontal cortex; F = female; IN = intranasal; IV = intravenous; M = male; mPFC = medial prefrontal cortex; ND = not detected; nM = nanomolar; NM = not measured; OFC = orbital frontal cortex; + = detected but not quantifiable.

a Material from: Lee and others (2020). Labeled oxytocin administered via the intranasal route reaches the brain in rhesus macaques, Nature Communications, published 2020 by Springer Nature under the terms of the Creative Commons CC BY license.

**Figure 2.**
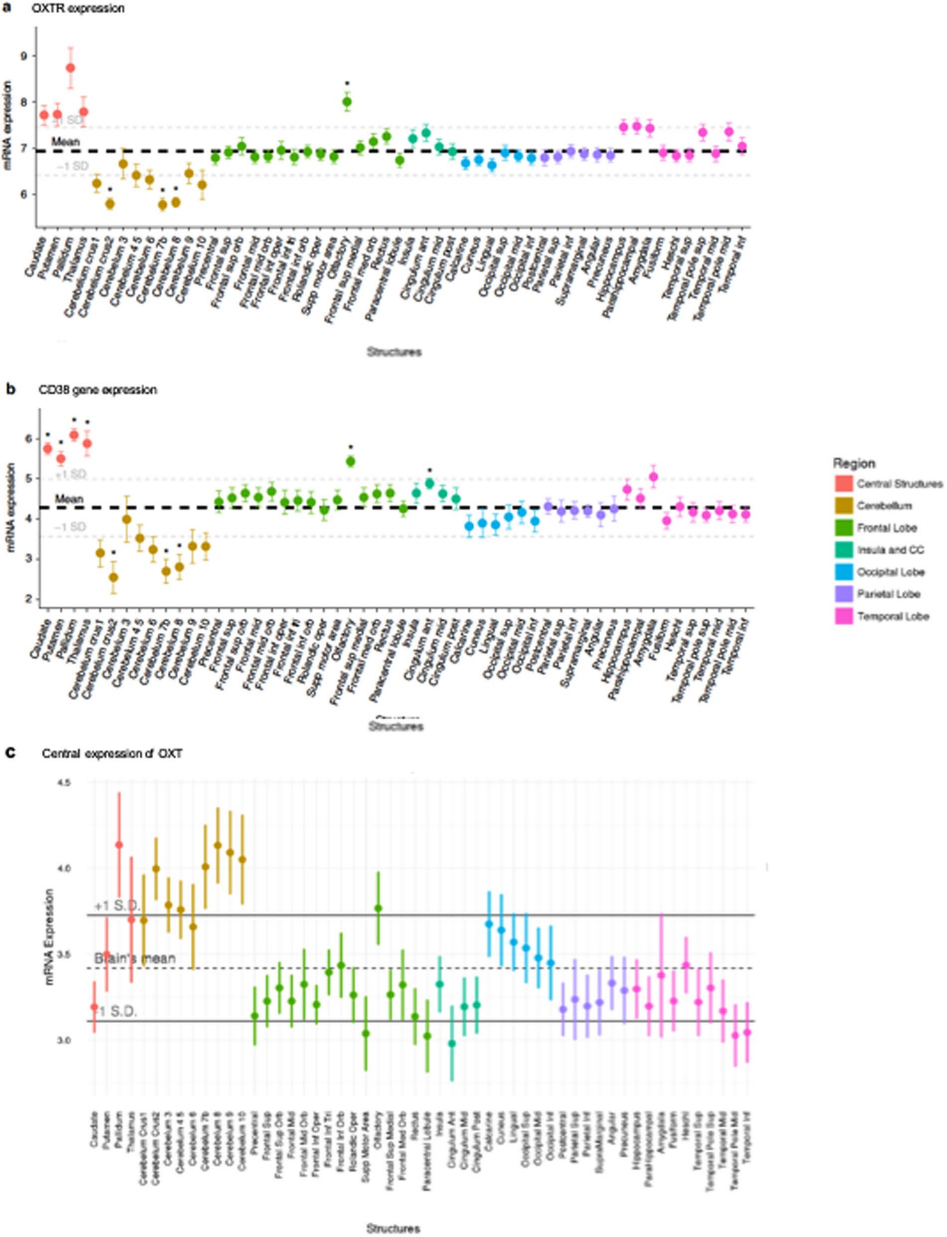
Oxytocin pathway gene expression in the postmortem human brain. Each point represents the mean expression from six donors collected from the Allen Human Brain Atlas (http://human.brain-map.org/). (a) Expression of OXTR mRNA. Higher than average expressions of OXTR mRNA levels was observed in the olfactory bulbs and several subcortical regions. (b) CD38 gene expression was higher than average in the caudate, pallidum, olfactory bulbs, putamen, thalamus, and cingulate anterior. (c) The central expression of OXT. No brain regions were associated with higher than average OXT expression after FDR correction. The bolded dashed lines represent mean expression across all regions plus 1 standard deviation (+/−). CD38, cluster of differentiation 38; FDR, false discovery rate; mRNA, messenger ribonucleic acid; OXT, oxytocin, OXTR, oxytocin receptor; SD, standard deviation. Material from: Quintana and others (2019). Oxytocin pathway gene networks in the human brain, Nature Communications, published 2019 by Springer Nature under the terms of the Creative Commons CC BY license.

Variation in the endogenous OXT system can be examined by genotyping OXT and related peptide and receptor polymorphisms. Although there are numerous studies reporting neurobehavioral differences as a function of genetic variation in OXT or its receptor, one significant limitation preventing assessment of questions regarding brain penetrance is the difficulty detecting OXTRs in primate species’ brains. However, with a competitive autoradiography binding method recently developed, the receptor was detected particularly in forebrain cholinergic regions of macaques (Freeman and others 2014). More recently, Rogers and colleagues (2018) used a mouse monoclonal antibody to find evidence for OXT-containing fibers in the cortex of humans and chimpanzees. Specifically, the researchers found OXT-immunoreactive fibers in the orbitofrontal cortex and anterior cingulate gyrus in both human and chimpanzee brains (but not those of rhesus macaques). In humans, a recent analysis of the Allen brain atlas data (Quintana and others 2019) reported greater than average OXTR mRNA concentrations in olfactory regions, striatum, and thalamus (Fig. 2), the same regions where Lee and colleagues (2020) have found evidence of receptor binding of labeled intranasally administered OXT.

### Methodological Implications for Human OXT Research

Most studies using intranasal OXT report subtle behavior-modifying effects with effect sizes ranging from weak (e.g., *d* = 0.21 for face recognition) to moderate (e.g., *d* = 0.43 for in-group trust) (Van IJzendoorn and Bakermans-Kranenburg 2012). This may be reflective of the relatively diffuse action of the peptide due to its role as neuromodulator. When administered at higher doses, or when added to heightened endogenous levels, OXT may interact with other hormonal and neurotransmitter systems, which could explain some of the inconsistencies of OXT effects observed in some experimental studies. For example, dose-response studies of OXT effects on amygdala reactivity in males have found that with the highest test dose (48 IU), amygdala reactivity was increased rather than decreased, suggesting a quadratic (inverted U-type) function of OXT action (Spengler and others 2017b), perhaps resulting from OXT binding to vasopressin receptors at higher central OXT concentrations.

Another factor strongly associated with heterogeneous responses to intranasal OXT is the peptide’s vulnerability to various contextual and psychosocial influences. OXT interacts with a variety of person-specific factors, particularly those related to baseline levels of interpersonal trust and affiliation. For example, Declerck and others (2020) found that OXT selectively increases trust in participants who have a low disposition to trust, Furthermore, OXT has been shown to increase altruistic donations, but only in participants who experienced low levels of parental love-withdrawal (Van IJzendoorn and others 2011). And in an in-group versus out-group setting, out-group-directed donations increased only in participants scoring low in xenophobia. In contrast, those with higher levels of xenophobia generally failed to exhibit enhanced altruism toward the out-group. This tendency was only countered by pairing OXT with peer-derived altruistic norms (Marsh and others 2017). More evidence indicating that it is important to consider person-specific differences when designing and interpreting OXT research comes from a study showing that the peptide increased cooperation and trust, and reduced betrayal aversion in participants scoring high on attachment avoidance, whereas these prosocial effects were absent in individuals scoring high on attachment anxiety (De Dreu 2012). The results of these studies suggest that variables associated with lower baseline trust and affiliation may increase the prosocial effects of exogenous OXT. However, other studies have shown that OXT decreases trust and prosocial behavior in patients with borderline personality disorder (Bartz and others 2011)—this may reflect the atypical and disorganized patterns of trust and affiliation observed in this disorder. Furthermore, OXT may promote prosocial behaviors and cognitions in the presence of social message frames (Marsh and others 2015) and when the social cues are interpreted as “safe” or “positive” (Declerck and others 2010; Olff and others 2013). Thus, it is essential to consider the role of both individual-level and contextual-level interpersonal and affective features in experimental studies using intranasal OXT (Insel 2016; Walum and others 2016), including the important role of “social message frames” (Hurlemann and Marsh, 2017).

Furthermore, OXT research and the interpretation of the data require highly controlled experimental environments and research protocols, for example, regarding dosage, sample size, task design, and timing. Current OXT-studies in humans often lack a comprehensive study-specific reporting of population characteristics, which are consistent across contexts. In addition to the preregistration of trials, which often involves the declaration of primary, secondary, and null effects in advance (Lane and others 2016; Leng and Ludwig 2016), the development of standardized sets of psychometric (self-)assessments for a given experimental context as well as analyzing and reporting those population characteristics may not only be a useful instrument to specify OXT effects within an experiment, but may also serve to increase the comparability of OXT-effects across studies. In the following section, we reflect on OXT’s modulatory role in human altruism by further emphasizing the peptide’s sensitivity to context-dependent effects on prosocial behavior.

## Oxytocin and the Neuroendocrine Architecture of Prosocial Behavior

The term “prosocial” is associated with a wide range of positive social behaviors, including trust, cooperation, care, empathy, and altruism—all of which are mainstays of forming and maintaining adaptive human social relationships. Each of these behaviors requires a balance between others’ and one’s own goals and needs, an ability without which human relationships would fail. However, generosity varies significantly across individuals and relationships. Factors known to moderate prosocial behavior include, for example, social closeness, prior learning history, the acute need of the beneficiary, and the individual ability to recognize and respond to emotional cues (Marsh 2018a).

Perhaps the quintessential prosocial behavior is altruism. Altruism is characterized by non-reciprocal prosocial acts which are aimed at improving the welfare of another individual at a personal cost to the altruist (Batson 2011; Fehr and Fischbacher 2003). Such behavior can be observed across species, usually among genetically related individuals but also among unrelated but socially close members of a social group to promote mutual safety and belonging (Marsh 2016a). Thus, altruism is deeply rooted in humans’ evolutionary past, suggesting it is supported by ancient neurochemical systems. Extensive research points to the OXT-system being involved in both the formation and maintenance of social relationships (Hurlemann and Scheele 2016; Walum and Young 2018), in part through its central involvement in orchestrating altruism and associated prosocial behaviors. For example, its potential to attenuate hypothalamic-pituitary-adrenal axis activity in response to social stressors resonates with evidence showing that intranasally administered OXT dampens amygdala reactivity toward social fear signals in male humans (Eckstein and others 2015) and macaques (Liu and others 2015), facilitates interpersonal trust (Baumgartner and others 2008; Kosfeld and others 2005), and promotes the formation and maintenance of interpersonal bonds (Insel 1997). However, the direction and magnitude of OXT’s prosocial effects are shaped by individual personality and environmental factors. In some contexts, OXT can also evoke aggressive-defensive responses and other antisocial behaviors, including conflict, prejudice, or envy (De Dreu and others 2011; Hurlemann and Marsh 2019; Shamay-Tsoory and others 2009). In addition to OXT’s sexually dimorphic effects (e.g., Gao and others 2016), these varying behavioral responses may result from individual variation in perceptions of social threat, social attitudes, and altered sensing of and responding to emotional stimuli (Guastella and others 2008; Spengler and others 2017a).

**Figure 3.**
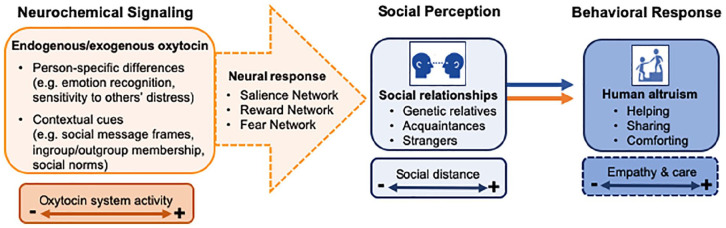
The influence of oxytocin on human altruism. The behavior-modifying effects of oxytocin (OXT) are highly sensitive to person-specific and context-related variables. According to our model, intranasal OXT modulates functioning in at least three partially overlapping neural networks, including salience-, reward-, and fear-related circuits. Thus, OXT-signaling in the brain is centrally involved in the formation and maintenance of social relationships that rely on empathy- and care-based mechanisms that facilitate altruistic behavior toward others. For example, OXT may promote an altruistic response bias toward a stranger in need by attenuating amygdala reactivity and increasing the perceived salience of social approach signals.

In the following sections, we provide a detailed overview of the modulatory role of OXT on of human altruism, particularly altruism emerging from evolved care-based mechanisms, which is largely motivated by empathy (Fig. 3). In addition, we reflect on OXT’s propensity to promote extreme forms of altruism, which may translate into hostile behaviors and enduring conflict.

### Care-Based Altruism

From an evolutionary perspective, altruistic care of vulnerable offspring is a necessity in mammalian species, who are born relatively altricial, dependent on milk for sustenance, and reliant on care for survival in habitats characterized by ubiquitous social and environmental threats (Krebs and Davies 1993). While OXT-like peptides lie at the core of the evolution of reproductive functions, OXT per se is exclusive to mammals, which suggests that it has evolved to support unique physiological and behavioral aspects of mammalian parental care (Hurlemann and Scheele 2016; Walum and Young 2018). In human and non-human mothers, changes in OXT production and receptor density during gestation promote care-based processes that continue throughout (and often beyond) the nursing period, whereas in fathers the care-based phase often is initiated at or after the time of birth (Marsh 2018a). The quality of both mothers’ and fathers’ infant care has been demonstrated to correspond to changes in peripherally measured OXT, whether due to endogenous changes in OXT concentration or increases following interactions with infants (Feldman and others 2010). Experimental evidence has shown that intranasal OXT administration also modulates neural responsiveness to infants in key parental care regions, including the amygdala and striatum, increases adults’ preferences for infant faces, promotes women’s responsiveness to infant crying (Marsh and others 2012; Riem and others 2011), and also improves the overall sensitivity of parental care and reduces hostility in fathers and motivates them to support exploration behavior in their children (Naber and others 2010). In response to child pictures, fathers furthermore exhibit higher OXT plasma concentrations as well as a stronger activation in brain regions important for the decoding of facial emotion (caudal middle frontal gyrus), mentalizing (temporoparietal junction), and reward processing (medial orbitofrontal cortex) relative to non-fathers (Mascaro and others 2014). These findings are consistent with studies suggesting that intranasal OXT facilitates the perception of social stimuli and promotes reciprocal social communication (Domes and others 2013; Maier and others 2019; Spengler and others 2017a).

Although OXT modulation of care systems likely originally evolved to support offspring care specifically, in many social species, OXT has also come to support care for a wider array of others, including the offspring of other group members (alloparental care) as well as unrelated juveniles and even non-juveniles exhibiting cues that signal vulnerability (Keebaugh and others 2015; Okabe and others 2017). This view is further substantiated by experimental research showing that OXT selectively increases care, generosity, and altruistic behavior as a function of person-specific differences, for example, empathy and social closeness (Jones and Rachlin 2006; Strang and others 2017) and contextual factors, for example, the experimental framing, cooperative versus competitive settings, or social versus nonsocial message frames (Marsh and others 2015; Xu and others 2019). Beyond care-based altruism that is directed toward close others, humans also frequently engage in altruistic behavior toward unrelated others, ranging from coworkers to complete strangers—and which can be reflected in, for example, monetary donations for the needy, humanitarian aid for refugees, or living organ donations for critically ill patients (Fellner and Schwartz 1971; Marsh 2018a). Altruism toward unrelated others can be promoted by a variety of proximate motivations. When directed toward members of the in-group, the motivation to advance and defend the collective fitness of the group can promote altruism. The group can include any peer group an individual identifies with, including sport teams, political parties, religious communities, or nations. Altruism aimed at promoting the good of social groups is parochial and yields in-group favoritism (Bernhard and others 2006) along with the tendency to protect the in-group against outside threats (Choi and Bowles 2007). Altruism can also be promoted by cues signaling vulnerability or distress, which can result in empathic concern. This form of empathy has been proposed as a powerful motive for altruism toward distressed and vulnerable strangers (Marsh 2018b; Preston and de Waal 2002).

Each of these altruism-promoting mechanisms has been linked to OXT, which promotes the salience of outcomes of the in-group such that, for example, increased endogenous OXT corresponds to increased empathic neural responses to the pain of in-group members (Levy and others 2016). Intranasally administered OXT can also increase sensitivity to others’ distress, including pain (Abu-Akel and others 2015) and expressions of fear (Leppanen and others 2017), the perception of which has been linked to increased compassion and generosity. These effects appear to be mediated by brain areas associated with interpreting and response to threats and aversive cues, including the amygdala, the anterior insula, and the anterior cingulate cortex (Hein and others 2016; Marsh and others 2014), which also have been identified as important loci of intranasal OXT action in humans (Bos and others 2015; Eckstein and others 2015; Rilling and others 2012; Scheele and others 2013) and animals (Burkett and others 2016). Variation in OXT responding in these structures across individuals and contexts likely explains the variable effects of OXT relevant to altruism, with, for example, in-group favoritism modifiable by social norms (Marsh and others 2017) and empathic pain sensitivity modifiable by the instructions to take the perspective of the self versus the other (Abu-Akel and others 2015).

Beyond altruism, a variety of other prosocial outcomes are also promoted by OXT. These include cooperation, trust, and defense of the in-group. In a seminal behavioral study, Kosfeld and others (2005) tested the modulatory effects of intranasal OXT among unrelated individuals using a trust game in which monetary exchanges were made between an investor (the participant) and an anonymous trustee either framed as a computer or an unfamiliar person. Only in the latter condition, the peptide increased the amount of money investors gave to the trustee. In a recent replication study by Declerck and others (2020) it was shown that OXT selectively increases trust in participants who have a low disposition to trust, which substantiates the relevance for a more nuanced understanding of the peptide’s susceptibility to person-specific differences and situational factors when conducting OXT research. Another study by Declerck and colleagues (2010) found that intranasal OXT increased cooperation when social information was provided, and subjects had prior contact with an interaction partner (Declerck and others 2014). In contrast, OXT has been shown to increase hostile behaviors toward the out-group under experimental conditions in which a prosocial framing was absent (De Dreu and others 2010; De Dreu and Kret 2016). These findings parallel studies of altruism in which message frames have been demonstrated to modify the degree to which intranasal OXT induced a shift in altruistic priorities by substantially increasing donations toward a social charity at the cost of an ecological charity project (Marsh and others 2015). Collectively, this empirical evidence suggests that altruism and related prosocial behaviors are orchestrated by individual perceptions of and variations in social closeness and empathy, which are influenced by OXT activity in the brain.

### Conflict

Altruistic and hostile behaviors are often viewed as diametrically opposed. In fact, due to variability in individual, motivational, and situational factors, aggression and conflict may not be exclusively linked to antisocial motivations, in the same way as altruism may not exclusively result from prosocial motivations (Hurlemann and Marsh 2019; Marsh 2018a). In-group favoritism represents a form of altruism, which may result in hostile behaviors toward the out-group or other manifestations of pathological altruism. In the context of social groups, in-group favoritism is associated with defense of others with the (altruistic) aim of protecting vulnerable others from harm. One prominent example across mammalian species is the protective defense of offspring, which is regulated by OXT activity (Rilling and Young 2014). In some cases, altruism may take on pathological forms that reflect extreme acts of selfless behavior with negative consequences to the self or innocent others, ranging from co-dependency to death (Oakley 2013). This behavior may emerge from unconditional in-group commitment, which can translate into extreme behaviors in some individuals who are willing to make sacrifices for their groups regardless of personal costs and consequences for others. As such, in-group versus out-group settings that may evoke defensive-aggressive behaviors can also be potentiated by OXT. Interestingly, the formation and maintenance of (prosocial) in-group alliances is often enforced by internalized social norms (Bernhard and others 2006) along with personally costly sanctions against defectors of these norms. In contrast to previous studies emphasizing the efficacy of social norms as a potential means of stabilizing altruistic cooperation (Fehr and Fischbacher 2004) and interpersonal trust (Xu and others 2019), which is associated with OXT signaling and in-group conformity (De Dreu and Kret 2016; Huang and others 2015; Radke and de Bruijn 2012; Stallen and others 2012), a recent study combined both interventions showing that an OXT-enforced norm compliance promoted an altruistic response bias toward out-groups, even in those individuals who exhibited more selfish decisions in the absence of these exogenous interventions (Marsh and others 2017). Importantly, exogenous triggers, such as the combination of a social norm and heightened OXT system activity, have the potential to promote extreme behaviors and enduring conflict with out-groups. Given this empirical background, we conclude that key contextual factors can influence whether prosocial motivation may spiral into conflict, long-term hostilities, or even warfare between groups, and that these behaviors likely are moderated by OXT signaling in the brain.

## Translating Oxytocin Neuroscience to the Clinic

**Figure 4.**
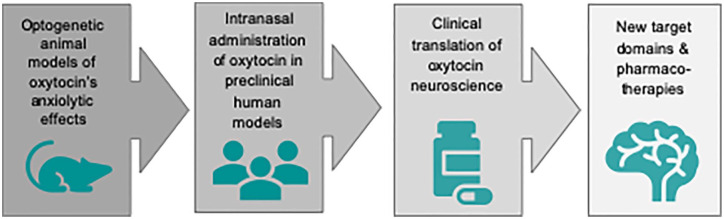
Translation of oxytocin neuroscience from bench to bedside. During the past decades there has been a growing interest in the therapeutic potential of oxytocin in psychiatric disorders, especially those characterized by social dysfunctions, which the available therapeutic compounds cannot fully target. Recent meta-analyses revealed a small effect size of oxytocin efficacy in schizophrenia and repetitive behaviors in autistic spectrum disorders (Peled-Avron and others 2020). From a mechanistic perspective, the prosocial effects of oxytocin seem particularly promising in clinical disorders of anxiety and antisociality (including psychopathy). Given oxytocin’s susceptibility to individual personality and situational variables, the clinical translation of oxytocin neuroscience to psychotherapy faces the crucial caveat that the therapeutic context should be strictly controlled.

There is emerging evidence that various psychiatric disorders marked by social dysfunction are correlated with dysregulation or malfunctioning of neuropeptidergic systems. The ability of OXT to modulate (pro-)social behavior has attracted increasing attention for translational preclinical and clinical investigations (Fig. 4).

Preliminary evidence has linked clinical disorders characterized by dysfunctions in adaptive social and prosocial behaviors to lack of OXT system activity, either under basal conditions, in response to social stimuli, or both. Schizophrenia, for example, is a condition marked by significant social dysfunction and high risk for antisocial outcomes, including paranoia (characterized by attributions of hostility and low social trust, among other features), low empathic accuracy, and increased reactive aggression (Savla and others 2013; Volavka and Citrome 2011). Several studies have now observed an inverse relationship between endogenous peripheral OXT levels and symptom severity in schizophrenic populations (Liu and others 2019; Rubin and others 2010). These findings have led to conjectures that increasing OXT might reduce symptom severity in this population. However, several trials have now sought to improve symptoms via exogenous oxytocin administered intravenously or intranasally and have generally not been successful (Bradley and Woolley 2017; Williams and Bürkner 2017), leading to suggestions that oxytocin—or at least acute levels of oxytocin—may not play a causal role in schizophrenic symptoms, or that the role of OXT may be complex and variable across individuals (Bradley and Woolley 2017).

Intranasal OXT has also been proposed as a potential treatment of anxiety disorders, following several studies demonstrating its anxiolytic effects. Currently, anxiety disorders are frequently treated with benzodiazepines, however, their clinical benefit is limited by side effects and addictive potential. Given its anxiolytic-like properties by inhibiting amygdala responses to fear signals in patients with anxiety disorders, OXT appears to be a potential new compound for anxiolytic drug development. A recent study tested the distinct anxiolytic mechanisms of OXT and the benzodiazepine Lorazepam using ultra-high-field (7-tesla) neuroimaging. It was found that OXT and lorazepam dampened responses to fear-related stimuli in the centromedial amygdala as a central hub of anxiolytic action, but only OXT induced large-scale connectivity changes of potential therapeutic relevance (Kreuder and others 2020).

More evidence supports the potential clinical efficacy of OXT in autistic spectrum disorders (ASD) (Parker and others 2017; Yatawara and others 2016). The possible link between ASD and oxytocin is supported by the fact that many of the key social deficits observed in ASD are related to outcomes linked to endogenous or exogenous increases in OXT levels in healthy populations. These include, for example, spontaneous attention to social stimuli like faces and eyes (Sasson and others 2007), empathic accuracy for nonverbal cues (Lozier and others 2014), and the perception of social stimuli as rewarding (Stavropolous and Carver 2014). This conclusion is also supported by single-dose functional brain imaging studies of individuals with ASD showing a task-related modulation of OXT on regional brain activity, which in some cases predicted improvements in social behavior, such as attention to social stimuli (Andari and others 2010) and empathy (Guastella and others 2010). Furthermore, efforts to improve performance in these domains often show the strongest effects among those with lower baseline performance. For example, several studies have now examined the effects of OXT on outcomes on the Reading the Mind in the Eyes Task—performance in which has been linked to autistic traits—and found greater improvement following OXT administration in participants with low baseline scores in trait empathy (Feeser and others 2015; Radke and de Bruijn 2015). Finally, as is the case for schizophrenia, reduced peripheral OXT levels have also been observed in ASD (Feldman and others 2014; Green and others, 2001). Although trials using continuous administration of OXT in subjects with ASD led to inconsistent findings (Young and Barrett 2015), there is evidence for OXT-induced improvements of social responsiveness in children with ASD (Parker and others 2017; Yatawara and others 2016). In the short term, OXT administration improves performance on several targeted tasks, including recognition of nonverbal emotion (Guastella and others 2010; Hollander and others 2007) and increased attention to and use of social cues during simulated interactions (Andari and others 2010). But efforts to maintain clinical improvements over time have been less successful. Although long-term treatment with intranasal OXT appears to be safe and well tolerated, improvements in social cognitive symptoms are typically small (Anagnostou and others 2014).

Attention has also been given to the potential clinical use of OXT for ameliorating antisocial behavior. Several lines of evidence point to a possible role for OXT in treating clinical disorders of antisociality, including psychopathy, antisocial personality disorder, and conduct disorder. Unlike schizophrenia and ASD, which are characterized by a range of both social and non-social symptoms (e.g., disordered thinking in schizophrenia, repetitive motor behaviors in ASD), disorders of antisociality are more narrowly characterized by their effects on social processes—and specifically prosocial processes. And although it should be emphasized that disorders of antisociality are orthogonal to other so-called disorders of empathy like ASD, individuals with both types of condition exhibit some common deficits in social cognition, including atypical social reward processing (Foulkes and others 2014), reduced attention to social cues (Boll and Gamer 2016; Dadds and others 2014a; Martin-Key and others 2018), and deficits in interpreting and responding to social cues. Unlike ASD, however, which impairs recognition of a wide range of nonverbal cues, disorders of antisociality particularly impair responses to nonverbal cues that convey distress, such as fearful facial expressions (Dawel and others 2012; Marsh and Blair 2008). This constellation of deficits is notable because they closely mirror the social cognitive domains most consistently associated with OXT administration, including increased salience of social rewards (Pfundmair and others 2017), increased attention to social cues (Hubble and others 2017), and—according to a recent meta-analysis—improved ability to recognize fearful facial expressions specifically (Leppanen and others 2017). This finding is noteworthy in light of evidence that increased sensitivity to fearful facial expressions (and other acute distress cues) is consistently associated with increased prosocial motivation and behavior (Marsh 2016b). Thus, there is indirect evidence that, despite OXT’s effects on prosocial behavior being complex, OXT could potentially improve outcomes for some of the core social cognitive deficits observed in disorders of antisociality.

Further supporting this possibility, various direct forms of evidence suggest functional alterations in the OXT system in disorders of antisociality (Rice and Derish 2015). Antisocial populations exhibit low peripheral OXT levels (Dadds and others 2014c; Levy and others 2015), possess genetic markers linked to reduced OXT functioning (Beitchman and others 2012; Dadds and others 2014b), and exhibit increased methylation of the OXT receptor gene, which is linked to lower *circulating* OXT (Dadds and others 2014c; Maud and others 2018). However, as yet no evidence demonstrates the possible efficacy of OXT in reducing clinically significant human antisociality. This lack of evidence may reflect unreported failures in clinical trials, although it may also reflect the extreme paucity of clinical research focusing on disorders of antisociality relative to ASD and schizophrenia. It should also be recalled that OXT increases some forms of aggression, primarily reactive and defensive aggression (e.g., maternal aggression; Ferris and others 1992), and has been linked to performance on laboratory tasks associated with increased aggression (Alcorn and others 2015; Ne’eman and others 2016)—although caution must be taken in generalizing from laboratory tasks to ecological forms of aggression. These findings, and the known differences in neurobiological mechanisms supporting divergent forms of aggression (Blair 2001), suggest that any potential clinical efficacy of OXT will likely be limited to specific contexts. Based on existing evidence, OXT’s effects on aggression would likely be strongest in the context of interactions with in-group members and/or the perception of salient distress cues expression by a victim, as sensitivity to these cues is enhanced by OXT and is associated with both reduced antisociality and increased prosociality. Collectively, the existing evidence emphasizes OXT’s potential as a new or adjunct compound for therapeutic use. However, given the peptides susceptibility to person-specific differences and contextual cues, future studies are warranted to investigate to what extent the clinical translation of OXT as a novel therapeutic agent requires the development of individually adjusted treatment strategies.

## Conclusion

OXT has a central role in modulating social behavior. Most human trials use intranasal OXT to experimentally explore the peptide’s effects on behavioral and neural outcome measures. In this conceptual and thematic overview, we reviewed evidence on nose-to-brain OXT administration as a valid method to experimentally model heightened endogenous OXT activity. In addition to the receptor distribution in different brain regions, OXT action is limited to a specific time window, the individual dose fraction reaching the brain after nasal delivery, and potential interactions with other hormonal and neurotransmitter systems. However, OXT-action in future studies might be improved by new methodologies, such as nanoparticle encapsulation (Oppong-Damoah and others 2019). Given the vulnerability of OXT effects to various individual and context-dependent influences, it is therefore essential to consider methodological standards in human OXT-studies, that is, adequate sample sizes and population characteristics, highly controlled study environments, and “message framing” within an experimental paradigm.

Insights to the modulatory role of OXT on human altruism have shown that the “framing” of external cues (e.g., social vs. environmental; positive in-group norms) shapes the magnitude and direction of OXT-effects. As such, OXT cannot be considered as a “designer drug” with a dose-linear mode of action but is a highly complex hormonal neuromodulator. Based on this neurohormonal profile, the potential clinical efficacy of OXT will likely be limited to specific contexts, such that translating the neuroscience of OXT into clinical outcomes faces the crucial caveat that a therapeutic context should be carefully controlled to minimize the risk of unfavorable outcomes.
